# Prevalence and prognostic significance of malnutrition risk in patients with pulmonary tuberculosis: A hospital-based cohort study

**DOI:** 10.3389/fpubh.2022.1039661

**Published:** 2022-12-13

**Authors:** Jiao-Jie Ma, Yi-Jia Guo, Zhuo Li, Yang Chen, Hong He, Wei-Min Li

**Affiliations:** ^1^Department of Nutrition, Beijing Chest Hospital, Capital Medical University, Beijing, China; ^2^Beijing Chest Hospital, Capital Medical University, Beijing, China; ^3^Beijing Municipal Key Laboratory of Clinical Epidemiology, School of Public Health, Capital Medical University, Beijing, China; ^4^National Tuberculosis Clinical Lab of China, Beijing Tuberculosis and Thoracic Tumor Research Institute and Beijing Key Laboratory in Drug Resistance Tuberculosis Research, Beijing, China

**Keywords:** pulmonary tuberculosis, malnutrition, cohort study, prevalence, prognostic

## Abstract

**Background:**

The prevalence and prognostic significance of malnutrition risk remain unclear in Chinese patients with pulmonary tuberculosis. Therefore, we aimed to investigate the malnutrition risk in Chinese patients and explore the relationship between malnutrition risk and follow-up outcomes.

**Methods:**

We conducted a hospital-based cohort study from January 2020 to December 2020. Malnutrition risks were evaluated using nutritional scales, including the Nutritional Risk Screening 2002 (NRS-2002), the controlling nutritional status score (CONUT), the geriatric nutritional risk index (GNRI), and the prognostic nutritional index (PNI). The primary outcome was all-cause mortality at a one-year follow-up. Malnutrition risk was calculated, and the relationship between malnutrition and follow-up outcomes was analyzed. We assessed the performance of malnutrition risks to predict clinical outcomes in prognostic models.

**Results:**

A total of 1,075 patients were included. According to NRS-2002, CONUT, GNRI, and PNI, 818 (76.09%), 954 (88.74%), 682 (63.44%), and 364 (33.86%) patients were at risk of malnutrition, respectively. Before 1-year follow-up, a total of 99 patients (9.2%) had died. After adjustment for risk factors, the association between severe malnutrition in CONUT (HR = 4.78, 95% CI: 1.14–20.11, *P* = 0.033), GNRI (HR = 3.53, 95% CI: 1.70–7.34, *P* = 0.001), or PNI (HR = 2.94, 95% CI: 1.76–4.88, *P* < 0.001) and death before 1-year follow-up remained significant. The addition of the nutritional scales to prognostic models improved death prediction, as validated by the integrated discrimination index (all *P-*values of <0.05).

**Conclusion:**

Malnutrition in patients with pulmonary tuberculosis was associated with an increased risk of all-cause death in the long-term follow-up. Our findings provided evidence for the use of admission nutrition screening in patients with pulmonary tuberculosis.

## Introduction

Tuberculosis (TB) was the leading cause of mortality worldwide from a single infectious disease, with 10 million new cases and 1.2 million deaths in 2019 (1). Malnutrition is a notable risk factor that leads to higher mortality in patients with TB (2–6). Moreover, malnutrition is an independent predictor for the reactivation of TB (7) and is considered an important, potentially reversible risk factor for treatment failure (8). However, nutritional status is often overlooked in patients with TB. The recently reported prevalence of malnutrition in patients with TB was estimated to be 50%-57% in different countries (2–6), while there are not sufficient data in China.

In 2013, the WHO published the first nutritional guideline for patients with TB. The guideline used the body mass index (BMI) as a tool for assessing nutritional status (9). The BMI, used as the only tool to estimate nutritional status, could not fully assess malnutrition (10). The Chinese Medical Association Parenteral and Enteral Nutrition Branch recommends nutritional risk screening 2002 (NRS-2002) as the preferred tool for assessing nutritional status (11). NRS-2002 has been used to assess malnutrition in patients with TB in a small sample study (11). However, the NRS-2002 could not reflect the severity of malnutrition, and the study is limited by a lack of objective and quantitative evaluation (11).

Recently, several objective scales have been used for assessing malnutrition, including the controlling nutritional status (CONUT) score (12), the geriatric nutritional risk index (GNRI) (13), and the prognostic nutritional index (PNI) (14). Previous studies investigated these nutritional scales in patients with cardiovascular disease (15–17), stroke (18), and cancer (19). These studies demonstrated that nutritional scales could be a good predictor of treatment endpoints (15–19). However, the effectiveness of objective scales on malnutrition screening in patients with TB remains unclear.

In recent years, several predictive models of TB mortality have been developed (20, 21). However, the nutritional scale was ignored in these predictive models. We speculated that adding nutritional scales to statistical models may increase their predictive performance. In the present study, we aimed to investigate malnutrition in Chinese patients and explore the relationship between nutritional status and TB outcome. Moreover, we further compared the predictive performance of malnutrition risk using different nutritional scales.

## Materials and methods

### Study design

This study was based on single-center, prospective data from January 2020 to December 2020. The inclusion criteria were patients diagnosed with pulmonary tuberculosis and those aged older than 18 years. The diagnosis of pulmonary TB had to be based on the WHO guidelines (22) and was validated by radiographic or etiological examination. We excluded any patient who met the following criteria: a diagnosis of non-tuberculous mycobacterial lung disease; a medical history of retreatment tuberculosis; a medical history of cancer or other end-stage diseases (23); a personal history of alcohol and substance abuse; and missing data on body height, weight, or other blood parameters used to calculate malnutrition risk. This study was approved by the ethics committee at our hospital, and all patients or their relatives signed written informed consent for study participation. The study protocol was reported in accordance with the “Strengthening the Reporting of Observational Studies in Epidemiology” (STROBE) guideline (24).

### Data collection

Demographic characteristics, body height or weight, and medical history were collected at baseline. Clinical features and nutrition status were evaluated by an experienced nutritionist from the department of nutrition in our hospital. Laboratory parameters were collected from a hospital-based database. Weight divided by height square [kg/m^2^] was used to calculate BMI.

The BMI categories were defined as follows: underweight (<18.5 kg/m^2^), normal weight (18.5–24.9 kg/m^2^), overweight (25.0–29.9kg/m^2^), and obesity (≥30.0 kg/m^2^). Baseline sputum samples were collected at admission, and sputum smears and drug-sensitive tests were performed at the laboratory in our hospital. Tuberculosis drug resistance was defined as resistance to at least one first-line anti-TB drug. All the laboratory parameters of the blood samples were obtained from the first-time results at admission.

### Nutritional screening scales and follow-up outcomes

The NRS-2002 is a routine scale performed in our hospital to identify patients with TB and malnutrition. The NRS-2002 scale included two main dimensions: impaired nutritional status and disease severity (23). A score between 0 and 3 was given for each dimension. Impaired nutritional status was calculated by three parameters: BMI, recent body mass loss, and food intake during the week before admission. Disease severity was calculated by evaluating the nutritional requirements caused by the medical history and concomitant chronic diseases. For patients over 70 years, an additional score of 1 point was added. The total NRS-2002 score is the sum of an impaired nutritional score, a severity of disease score, and an age score. The total scores range from 0 to 7. Patients with a score of ≥3 were considered to be malnutritioned and were recommended nutritional support (23).

We also explored other objective scales in patients with TB to investigate malnutrition, including the CONUT, GNRI, and PNI. To assess malnutrition risk, the CONUT included three parameters (total lymphocyte count, serum albumin, and cholesterol). The total scores ranged from 0 to 12, and malnutrition assessed by CONUT was defined as follows: normal (score 0–1), mild risk (score 2–4), moderate risk (score 5–8), and severe risk (score 9–12) (12).

The GNRI included three parameters (present body weight, ideal body weight, and serum albumin), and the score was calculated as (41.7×present weight [kg]/ideal body weight [kg]+ 1.519×serum albumin [g/L]). We calculated ideal body weight based on the following formula: for women, ideal body weight = height in cm – 100 – ([height in cm – 150]/2); and for men, ideal body weight = height in cm – 100 – ([height in cm−150]/4); malnutrition assessed by GNRI were defined as follows: normal (score >98), mild risk (score 92–98), moderate risk (score 82–91) and severe risk (score<82) (13).

The PNI included two parameters (total lymphocyte count and serum albumin), and the score was calculated using the formula: 0.005×total lymphocyte count (mm^3^) + 10×serum albumin (g/dl). Malnutrition assessed by PNI was defined as follows: normal (score>38), moderate risk (score 35–38), and severe risk (score <35) (14).

A face-to-face follow-up or telephone interview was performed 1 year after admission. We obtained follow-up information on all-cause death from relatives of patients or a death certificate from our hospital records.

### Statistical analyses

Descriptive characteristics were reported as percentages for categorical variables or as the mean with standard deviation for continuous variables χ^2^ test, Fisher exact test, Student's *t-*test, or the Mann–Whitney U-test were performed for statistical analysis whenever deemed appropriate. The malnutrition risk was assessed using the nutritional scales. Cox regression was used to investigate essential factors of mortality at a 1-year follow-up. The following covariates were adjusted in the Cox regression model: age, gender, hypertension, coronary heart disease, chronic obstructive pulmonary disease, meningeal tuberculosis, and tuberculosis drug resistance (*P* < 0.1 by univariate analysis or clinical confounders). We calculated the net reclassification improvement (NRI) and integrated discrimination improvement (IDI) to quantify the correct reclassification and sensitivity improvement with the addition of nutritional scales in the predictive model. Sensitivity analyses were performed by different adjusted models. Significant improvement was recognized in the prediction model when NRI >0 or IDI >0 (24). All tests were 2-tailed, and a *P-*value <0.05 was considered statistically significant. All analyses were conducted using R version 4.2.0.

## Results

### Baseline characteristics

**Figure 1:**
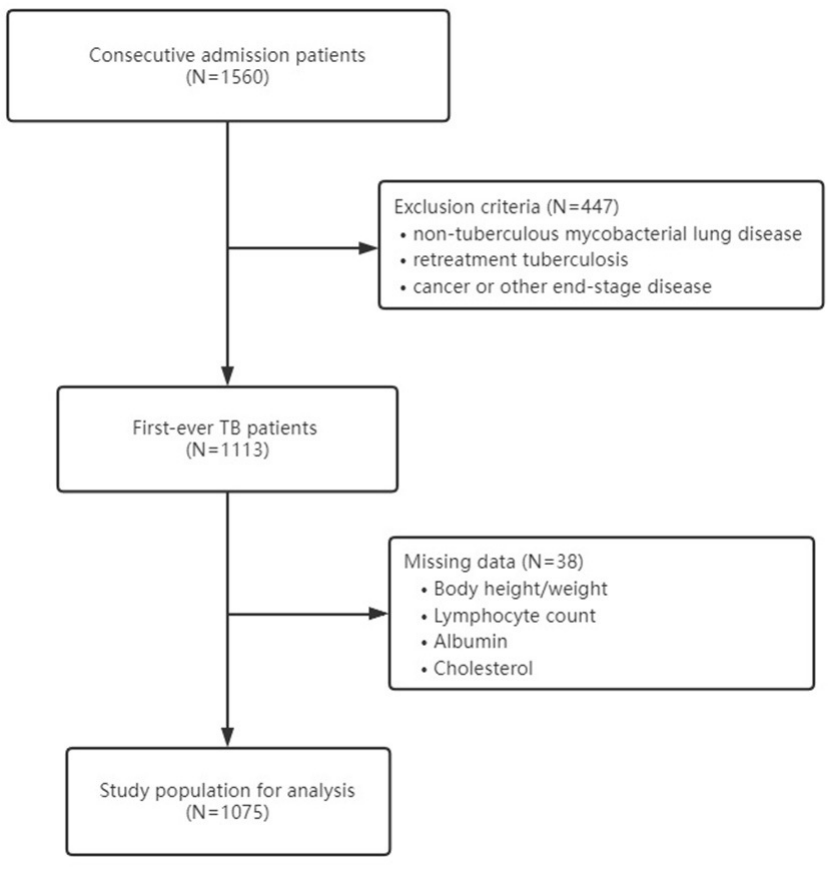
Study flow chart.

At baseline, there were 1,560 consecutive in-hospital Chinese patients enrolled in the cohort. After excluding patients with non-tuberculous mycobacterial lung disease (*n* = 46), patients with a medical history of retreatment tuberculosis (*n* = 392), patients with cancer or other end-stage diseases (*n* = 9), and patients with missing data on body height, weight, or other parameters used to calculate malnutrition (*n* = 38), a total of 1,075 patients were analyzed in the study (Figure 1). The mean age was 52.73 (±20.71) years, and 63.16% were men. The baseline information of study participants is shown in Table 1.

**Table 1:** Baseline information of study participants stratified by nutritional status using NRS-2002.

**Variables**	**Overall** **(*n =* 1,075)**	**With Malnutrition** **(*n =* 818)**	**Without Malnutrition** **(*n =* 257)**	***P*** **-value**
**Demographics**				
Age, y	52.73 ± 20.71	55.12 ± 21.38	45.14 ± 16.23	<0.001
Men	679 (63.16)	521(63.69)	158(61.48)	0.521
Height, cm	167.80 ± 8.30	167.55 ± 8.29	168.59 ± 8.31	0.078
Weight, kg	60.24 ± 10.45	58.02 ± 9.42	67.30 ± 10.42	<0.001
Body mass index, kg/m^2^	21.33 ± 3.01	20.61 ± 2.81	23.60 ± 2.45	<0.001
**Medical history**				
Hypertension	153(14.23)	119(14.55)	34(13.23)	0.598
Diabetes	272(25.30)	211(25.79)	61(23.74)	0.508
Coronary heart disease	115(10.69)	96(11.74)	19(7.39)	0.049
Stroke	14(1.30)	13(1.59)	1(0.3)	0.208
Chronic obstructive pulmonary disease	19(1.76)	16(1.96)	3(1.67)	0.588
**Clinical features**				
Military TB	32(2.97)	25(3.06)	7(2.72)	0.784
Meningeal TB	10(0.93)	8(0.98)	2(0.78)	0.971
Positive sputum smear	306(28.46)	261(31.91)	45(17.51)	<0.001
TB drug-resistant	132(12.27)	96(11.74)	36(14.01)	0.333
HIV infection	1(0.10)	1(0.10)	0(0.00)	1.000
Hospital stay	15.99 ± 12.38	16.85 ± 13.10	13.24 ± 9.21	<0.001
**Laboratory feature**				
Hemoglobin, g/L	120.49 ± 22.30	115.06 ± 21.64	137.75 ± 14.09	<0.001
Lymphocyte, 10^9^/L	1.28 ± 0.60	1.18 ± 0.59	1.58 ± 0.53	<0.001
Urea, mmol/l	4.80 ± 2.68	4.97 ± 2.98	4.28 ±1.20	<0.001
Creatinine,μmol/L	64.51 ± 33.29	64.82 ± 37.33	63.53 ± 14.18	0.586
Uric acid, μmol/L	327.86 ± 144.59	311.73 ± 143.85	379.16 ± 134.91	<0.001
Albumin, g/L	35.02 ± 6.94	33.16 ± 6.74	40.98 ± 3.20	<0.001
Cholesterol, mmol/L	4.22 ± 0.86	4.14 ± 0.81	4.52 ± 0.92	<0.001
Triglycerides, mmol/L	1.28 ± 0.58	1.24 ± 0.53	1.40 ± 0.69	<0.001

Continuous variables are expressed as mean±SD, and categorical variables are expressed as frequency (%). TB, tuberculosis; HIV, human immunodeficiency virus.

### Malnutrition risk according to clinical nutrition scales

**Figure 2:**
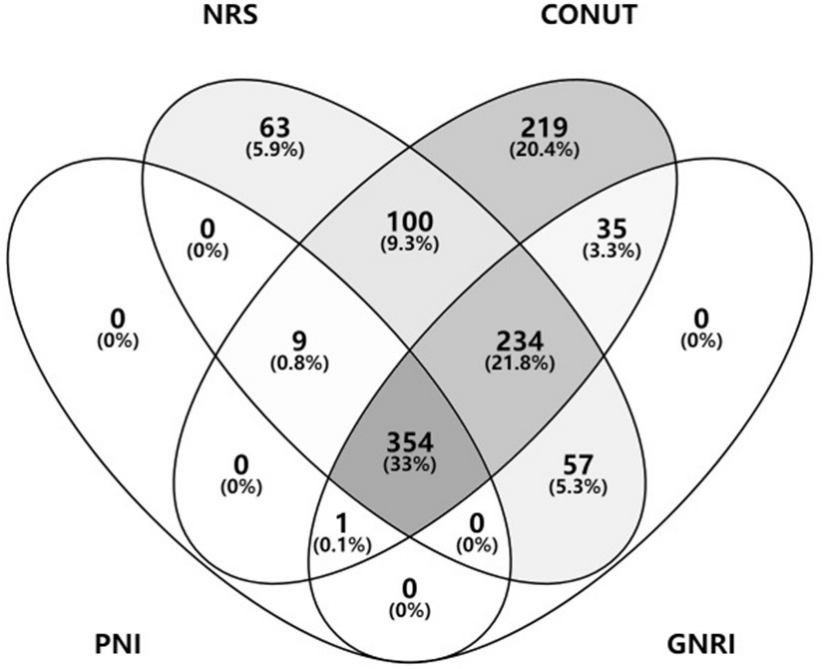
Malnutrition risk assessed by the nutritional scales. BMI, body mass index; NRS, nutritional risk screening; CONUT, controlling nutritional status; GNRI, geriatric nutritional risk index; PNI, prognostic nutritional index.

According to NRS-2002, CONUT, GNRI, and PNI, 818 (76.09%), 954 (88.74%), 682 (63.44%), and 364 (33.86%) patients were at risk of malnutrition (Table 2), whereas 167 (15.53%) patients had malnutrition, as assessed by the underweight BMI. Venn diagram showed malnutrition risk assessed by the 4 nutritional scales (Figure 2).

**Table 2:** The prevalence of the malnutrition risk identified by the nutritional scales.

	**Malnutrition risk**				
	**Normal, %**	**Any**	**Mild**	**Moderate**	**Severe**
BMI	73.02(70.24–75.63)	15.53(13.44–17.87)		…	…
NRS2002	23.91(21.41–26.60)	76.09(73.40–78.59)	…	…	…
CONUT	11.25(9.46–13.34)	88.74(86.66–90.54)	29.48(26.80–32.33)	37.95(35.05–40.94)	21.30(18.91–23.90)
GNRI	36.55(33.69–39.53)	63.44(60.47–66.31)	17.39(15.21–19.83)	24.55(22.04–27.27)	21.49(19.09–24.09)
PNI	66.13(63.21–68.95)	33.86(31.05–36.79)	…	8.93(7.33–10.83)	24.93(22.39–27.65)

BMI, body mass index; NRS, nutritional risk screening; CONUT, controlling nutritional status; GNRI, geriatric nutritional risk index; PNI, prognostic nutritional index.

A more detailed stratified analysis of the severity of malnutrition was performed. Of these patients, 317 (29.48%) and 187 (17.39%) were at mild malnutrition risk, as assessed by CONUT and GNRI; 408 (37.95%), 264 (24.55%), and 96 (8.93%) patients were at moderate malnutrition risk, as assessed by CONUT, GNRI, and PNI; and 229 (21.30%), 231 (21.49%), and 268 (24.93%) patients were at severe malnutrition risk, as assessed by CONUT, GNRI, and PNI, respectively.

### Predictors of follow-up clinical events

Before 1-year follow-up, a total of 99 patients (9.2%) had died. Univariable analyses suggested that malnutrition was significantly associated with follow-up death (Supplementary Table 1). Any malnutrition assessed by the NRS-2002, GNRI, or PNI and moderate to severe malnutrition assessed by CONUT was related to an increased risk of death at 1-year follow-up (Table 3). In multivariable analyses, after adjustment for age, gender, hypertension, coronary heart disease, chronic obstructive pulmonary disease, meningeal tuberculosis, and tuberculosis drug resistance, the association between severe malnutrition in CONUT (HR = 4.78, 95% CI 1.14–20.11, *P* = 0.033), GNRI (HR = 3.53, 95% CI 1.70–7.34, *P* = 0.001), or PNI (HR = 2.94, 95% CI 1.76–4.88, *P* < 0.001) and death before 1-year follow-up remained significant.

**Table 3:** Multivariable analyses of malnutrition scales to predict 1-year mortality.

**Nutritional status**	**Events**,	**Unadjusted**	**Model 1**	**Model 2**	**Model 3**
	***N*** **(%)**	**HR (95% CI)**	**HR (95% CI)**	**HR (95% CI)**	**HR (95% CI)**
**BMI**					
Underweight	6(6.00)	0.32(0.14–0.73)^*^	0.51(0.22–1.16)	0.53(0.23–1.21)	0.56(0.24–1.28)
Normal	88(88.80)	Reference	Reference	Reference	Reference
Overweight-Obesity	5(5.00)	0.36(0.15–0.89)^*^	0.46(0.18–1.16)	0.49(0.19–1.22)	0.50(0.20–1.26)
**NRS2002**					
Normal	5(5.10)	Reference	Reference	Reference	Reference
Any risk	94(94.90)	2.78(2.16–3.58)^*^	2.66(1.06–6.70)^*^	2.61(1.04–6.59)^*^	2.45(0.97–6.22)
**CONUT**					
Normal	2(2.00)	Reference	Reference	Reference	Reference
Mild risk	9(9.00)	1.72(0.37–7.95)	1.76(0.38–8.18)	1.76(0.38–8.17)	1.46(0.31–6.86)
Moderate risk	32(32.30)	4.75(1.14–19.80)^*^	2.64(0.63–11.09)	2.60(0.62–10.98)	2.41(0.57–10.18)
Severe risk	56(56.50)	14.79(3.61–60.63)^*^	5.29(1.27–22.16)^*^	4.92(1.17–20.67)^*^	4.78(1.14–20.11)^*^
**GNRI**					
Normal	10(10.10)	Reference	Reference	Reference	Reference
Mild risk	12(12.10)	2.51(1.08–5.81)^*^	1.76(0.75–4.09)	1.76(0.76–4.13)	1.45(0.60–3.49)
Moderate risk	25(25.20)	3.72(1.78–7.75)^*^	2.01(0.94–4.25)	1.85(0.86–3.94)	1.98(0.92–4.28)
Severe risk	52(52.50)	8.88(4.52–17.48)^*^	3.42(1.68–7.01)^*^	3.31(1.61–6.81)^*^	3.53(1.70–7.34)^*^
**PNI**					
Normal	25(25.20)	Reference	Reference	Reference	Reference
Moderate risk	9(9.00)	2.66(1.25–5.71)^*^	1.45(0.67–3.15)	1.42(0.65–3.08)	1.48(0.68–3.21)
Severe risk	65(65.60)	6.89(4.35–10.94)^*^	3.02(1.83–4.99)^*^	2.85(1.71–4.75)^*^	2.94(1.76–4.88)^*^

Model 1: adjust for age, gender; Model 2: adjust for age, gender+hypertension, coronary heart disease, chronic obstructive pulmonary disease; Model 3: adjust for age, gender, hypertension, coronary heart disease, chronic obstructive pulmonary disease+ Meningeal TB, TB drug-resistant. BMI, body mass index; NRS, nutritional risk screening; CONUT, controlling nutritional status; GNRI, geriatric nutritional risk index; PNI, prognostic nutritional index. TB, tuberculosis; HR, hazard ratio.

* P < 0.05.

### Improvement in models upon the addition of adding clinical nutrition scales

We investigated the performance of models to predict clinical outcomes in patients with TB (Table 4). The predictive performance, according to the C-statistic, slightly improved when clinical nutrition scales were added to the different adjusted models. Nutrition scales improved the model's performance, as confirmed by NRI and IDI. The increased NRI ranged from 3.92 to 24.43% in Model 1, from 3.73 to 22.43% in Model 2, and from 3.32 to 13.34% in Model 3, which suggested that the addition of nutrition scales improved the model's performance for predicting death. In the final Model 3, the NRI of CONUT, GNRI, and PNI was 9.40% (−0.02 to 18.82), 10.60% (0.85–20.35), and 13.34% (2.52–24.16), respectively. The improved performance of the model validated by IDI can likewise be interpreted as the explanation of NRI. The addition of BMI and nutrition scales to different adjusted models significantly improved predictive ability (all *P* < 0.05).

**Table 4:** Performance of prognostic models with malnutrition scales to predict the 1-year mortality in patients with TB.

	**C-statistic**		**Category NRI**		**IDI**	
	**Estimate (95% CI)**	***P*** **-value**	**Estimate (95% CI),%**	***P*** **-value**	**Estimate (95% CI),%**	***P*** **-value**
Model 1	0.8244 (0.7787–0.8700)	Reference	Reference		Reference	
Model+BMI	0.8314 (0.7868–0.8759)	0.183	3.92(−3.28 to 11.12)	0.286	1.01(0.36 to 1.65)	0.002
Model+NRS2002	0.8297 (0.7861–0.8732)	0.290	3.51(−0.01 to 7.94)	0.119	0.46(0.00 to 0.83)	0.016
Model+CONUT	0.8428 (0.7985–0.8871)	0.032	18.77(8.54 to 28.99)	<0.001	3.35(1.99 to 4.72)	<0.001
Model+GNRI	0.8392 (0.7946–0.8835)	0.073	20.89(10.45 to 31.33)	<0.001	2.62(1.31 to 3.92)	<0.001
Model+PNI	0.8434 (0.7985–0.8883)	0.038	24.43(13.29 to 35.58)	<0.001	4.24(2.67 to 5.81)	<0.001
Model 2	0.8334 (0.7877–0.8792)	Reference	Reference		Reference	
Model+BMI	0.8391 (0.7942–0.8840)	0.246	7.48 (−0.13 to 15.09)	0.054	0.91(0.32 to 1.50)	0.002
Model+NRS2002	0.8379 (0.7939–0.8819)	0.365	3.73 (−1.11 to 8.58)	0.131	0.57(0.19 to 0.95)	0.003
Model+CONUT	0.8480 (0.8035–0.8926)	0.056	21.31(11.38 to 31.25)	<0.001	2.83(1.59 to 4.08)	<0.001
Model+GNRI	0.8453 (0.8020–0.8885)	0.079	19.09 (8.99 to 29.18)	<0.001	2.39(1.16 to 3.61)	<0.001
Model+PNI	0.8491 (0.8043–0.8939)	0.066	22.43(11.58 to 33.27)	<0.001	3.71(2.23 to 5.19)	<0.001
Model 3	0.8592 (0.8221–0.8961)	Reference	Reference		Reference	
Model+BMI	0.8592 (0.8226–0.8962)	0.858	7.48(0.99 to 13.97)	0.0239	1.06(0.53 to 1.59)	<0.001
Model+NRS2002	0.8611 (0.8250–0.8971)	0.660	3.32(−1.55 to 8.19)	0.181	0.59(0.11 to 1.08)	0.015
Model+CONUT	0.8713 (0.8357–0.9070)	0.108	9.40(−0.02 to 18.82)	0.051	1.73(0.61 to 2.84)	0.002
Model+GNRI	0.8712 (0.8364–0.9059)	0.119	10.60(0.85 to 20.35)	0.033	1.84(0.67 to 3.01)	0.002
Model+PNI	0.8744 (0.8387–0.9100)	0.070	13.34(2.52 to 24.16)	0.016	2.33(1.01 to 3.66)	0.001

Model 1: adjusted for age, gender; Model 2: adjusted for age, gender+hypertension, coronary heart disease, and chronic obstructive pulmonary disease; Model 3: adjusted for age, gender, hypertension, coronary heart disease, chronic obstructive pulmonary disease+ Meningeal TB, and TB drug-resistance.

NRI, net reclassification improvement; IDI, integrated discrimination Improvement; BMI, body mass index; NRS, nutritional risk screening; CONUT, controlling nutritional status; GNRI, geriatric nutritional risk index; PNI, prognostic nutritional index. TB, tuberculosis.

## Discussion

The present study investigated the nutritional status of Chinese patients with TB and explored the performance of nutritional scales to predict TB outcomes. The findings suggested that severe malnutrition risk ranged from 21.30 to 24.93% in patients with TB. Baseline malnutrition may be a predictor of TB mortality. The addition of nutritional scales to mortality models improved C statistics, NRI, and IDI. The results stress the importance of assessing the nutritional status of patients with TB.

Our data showed that malnutrition risk ranged from 33.86 to 88.74% in patients with TB, the moderate risk was 8.93 to 37.95%, and the severe risk was 21.30 to 24.93%. The 2013 WHO guideline considers BMI as a tool for assessing malnutrition in patients with TB (9). However, in our data, only 15.53 % of individuals who are malnourished used BMI. Malnutrition was underestimated when compared to other nutritional scales. The previous study indicated that patients with catabolic diseases such as TB might be malnourished but still show a BMI between or above the normal range (25). This could be explained by the fact that BMI is a characteristic of chronic malnutrition that involves weight loss (26), whereas disease-associated malnutrition is a subacute or acute condition in which weight loss does not lead to a low BMI (26, 27). These results indicated that patients with TB would not be identified as malnourished when assessing nutritional status only based on BMI. The NRS-2002 is often used to assess for malnutrition in Chinese patients (28). It is an important tool for assessing Chinese patients with malnutrition and whether they need nutritional intervention (29). A recent study confirmed that malnutrition was assessed by the NRS-2002 in Chinese patients with TB with a prevalence of 64.41% (11), which was similar to the results. However, the NRS-2002 is limited by potential subjective bias and cannot quantitatively evaluate the severity of malnutrition.

Therefore, we compared the performance of other objective nutritional scales in our study. The malnutrition risk varied between different objective scales. The malnutrition risk calculated by CONUT was 88.74%, whereas 63.44% was calculated by GNRI and 33.86% by PNI. The GNRI (13) involves weight and serum albumin parameters, and the CONUT (12) includes serum albumin, lymphocyte count, and total cholesterol level. Compared to the CONUT, the PNI (14) only includes the parameters of albumin and lymphocyte count but lacks the parameter of cholesterol level, which may explain the lower prevalence estimated by the PNI. Nevertheless, the prevalence of severe malnutrition risk assessed by objective scales ranged from 21.30 to 24.93% in patients with TB, which suggested good internal consistency. Based on the literature review and findings from our study, PNI may be a more suitable scale to screen for malnutrition risk in patients with TB.

Several studies examined the association between nutritional status and TB mortality (2, 4). A prospective cohort of 1,695 adult patients with pulmonary TB was evaluated for malnutrition risk using the BMI, and the results showed that severe undernutrition was associated with a two-fold higher risk of death (2). In another study, nutritional status, also assessed by the BMI, in 1,181 patients with TB revealed that moderate to severe malnutrition is a risk factor associated with early death (4). The correlation between malnutrition and TB mortality was also verified by the Malnutrition Screening Tool (MST) (30), the Mini Nutritional Assessment (MNA) (31), and the Malnutrition Universal Screening Tool (MUST) (32) in small sample size studies (30–34). However, few studies reported the effect of objective scales on nutritional screening for patients with TB. In the present study, we found that severe malnutrition risk evaluated by objective nutritional scales was significantly associated with mortality after adjusting for the potential risk factors. Therefore, physicians should consider on-admission nutritional screening for patients with TB.

Several prognostic models have been developed to predict TB mortality. However, nutritional status was not involved in these studies (20, 21). We added nutritional scales to the statistical models to predict TB outcomes. The predictive ability of nutritional scales for mortality was improved and validated by increased C-statistic, NRI, and IDI. Our study demonstrated that objective nutritional scales might improve malnutrition risk classification for TB mortality.

Our study has its limitations. First, due to the unavailability of necessary variables, we failed to investigate malnutrition according to the European diagnostic criteria (26) and could not perform a comparison with other nutritional screening tools such as MST (30), MNA (31), and MUST (32). However, there is currently no gold standard for nutritional screening in patients with TB. The WHO guidelines only recommend BMI as a tool for assessing nutritional status. Future studies should investigate nutritional status using more evaluation methods. Second, the study only evaluated admission nutritional status—not at follow-up. We were unable to consider dynamic nutritional changes that may affect TB outcomes. Third, the follow-up period in our study was not long enough, and only the death rate 1 year after admission was followed up, which may not be sufficient for evaluating TB outcomes. Finally, we excluded several patients whose data were incomplete, which may result in potential selection bias. Multicenter studies with large sample sizes are required to generalize these findings.

In conclusion, severe malnutrition risk ranged from 21.30 to 24.93% in Chinese patients with TB. Malnutrition risk was related to an increased risk of death in the long-term follow-up. Nutritional scales may be significant indicators for predicting clinical outcomes in patients with TB.

## Data availability statement

The raw data supporting the conclusions of this article will be made available by the authors, without undue reservation.

## Ethics statement

The studies involving human participants were reviewed and approved by the Ethics Committee at Beijing Chest Hospital. The patients/participants provided their written informed consent to participate in this study.

## Author contributions

Conceptualization: J-JM and W-ML. Data collection: J-JM, ZL, YC, and HH. Formal analysis: Y-JG. Funding acquisition: W-ML. Methodology: Y-JG and W-ML. Data supervision: J-JM and W-ML. Writing—original draft: J-JM and Y-JG. Writing—review and editing: J-JM, Y-JG, ZL, YC, HH, and W-ML. All authors contributed to the article and approved the submitted version.
