# Supplementary material for: Prevalence and prognostic significance of malnutrition risk in patients with pulmonary tuberculosis: A hospital-based cohort study
Source: Front Public Health. 2022 Dec 13;10:1039661. doi: 10.3389/fpubh.2022.1039661 (PMC9792975; doi:10.3389/fpubh.2022.1039661)
Supplement: Supplementary file 1 [file Table_1.docx]

**Supplementary Table. Univariable Analyses of Risk Factors for 1-Year Mortality**

| Variable | HR (95%CI) | P-value |
| --- | --- | --- |
| **Demographics** |  |  |
| Age, y | 1.06(1.05-1.08) | <0.001 |
| Men | 1.56(0.99-2.42) | 0.050 |
| Height, cm | 0.98(0.96-1.01) | 0.155 |
| Weight, kg | 0.99(0.98-1.02) | 0.765 |
| Body mass index, kg/m^2^ | 1.00(0.94-1.07) | 0.946 |
| **Medical history** |  |  |
| Hypertension | 1.82(1.14-2.91) | 0.012 |
| Diabetes | 0.99(0.63-1.57) | 0.991 |
| Coronary heart disease | 1.61(0.94-2.75) | 0.081 |
| Stroke | 1.56(0.39-6.34) | 0.532 |
| Chronic obstructive pulmonary disease | 4.23(1.96-9.12) | <0.001 |
| **Clinical features** |  |  |
| Military TB | 1.37(0.51-3.73) | 0.535 |
| Meningeal TB | 4.48(1.65-12.19) | 0.003 |
| Smear positive | 1.09(0.71-1.68) | 0.685 |
| TB drug-resistant | 0.54(0.25-1.17) | 0.120 |
| Hospital stay | 1.01(0.99-1.02) | 0.114 |
| **Laboratory feature** |  |  |
| Hemoglobin, g/L | 0.98(0.97-0.99) | <0.001 |
| Lymphocyte, 10^9^/L | 0.33(0.22-0.49) | <0.001 |
| Urea, mmol/l | 1.14(1.09-1.18) | <0.001 |
| Creatinine,μmol/L | 1.01(1.00-1.01) | 0.001 |
| Uric acid, μmol/L | 0.99(0.99-1.00) | 0.045 |
| Albumin, g/L | 0.88(0.86-0.90) | <0.001 |
| Cholesterol, mmol/L | 0.89(0.71-1.13) | 0.338 |
| Triglycerides, mmol/L | 1.06(0.76-1.47) | 0.742 |
| **Nutritional status** |  |  |
| **BMI** |  |  |
| Underweight | 0.32(0.14-0.73) | 0.007 |
| Normal | Reference | Reference |
| Overweight-Obesity | 0.36(0.15-0.89) | 0.027 |
| **NRS2002** |  |  |
| Normal | Reference | Reference |
| Any risk | 5.91(2.40-14.52) | <0.001 |
| **CONUT** |  |  |
| Normal | Reference | Reference |
| Mild risk | 1.72(0.37-7.95) | 0.489 |
| Moderate risk | 4.75(1.14-19.80) | 0.033 |
| Severe risk | 14.79(3.61-60.62) | <0.001 |
| **GNRI** |  |  |
| Normal | Reference | Reference |
| Mild risk | 2.51(1.08-5.81) | 0.032 |
| Moderate risk | 3.72(1.79-7.75) | <0.001 |
| Severe risk | 8.89(4.52-17.48) | <0.001 |
| **PNI** |  |  |
| Normal | Reference | Reference |
| Moderate risk | 2.66(1.25-5.71) | 0.012 |
| Severe risk | 6.89(4.35-10.94) | <0.001 |

TB,tuberculosis; BMI, body mass index; NRS, nutritional risk screening; CONUT, controlling nutritional status;

GNRI, geriatric nutritional risk index; PNI, prognostic nutritional index.
